# Prototype Machine Learning Algorithms from Wearable Technology to Detect Tennis Stroke and Movement Actions

**DOI:** 10.3390/s22228868

**Published:** 2022-11-16

**Authors:** Thomas Perri, Machar Reid, Alistair Murphy, Kieran Howle, Rob Duffield

**Affiliations:** 1 School of Sport, Exercise and Rehabilitation, Faculty of Health, University of Technology Sydney, Ultimo, NSW 2007, Australia; 2 Tennis Australia, Melbourne, VIC 3000, Australia; 3 Catapult Sports, Melbourne, VIC 3000, Australia

**Keywords:** wearable technology, machine learning, accelerometery, racquet sports

## Abstract

This study evaluated the accuracy of tennis-specific stroke and movement event detection algorithms from a cervically mounted wearable sensor containing a triaxial accelerometer, gyroscope and magnetometer. Stroke and movement data from up to eight high-performance tennis players were captured in match-play and movement drills. Prototype algorithms classified stroke (i.e., forehand, backhand, serve) and movement (i.e., “Alert”, “Dynamic”, “Running”, “Low Intensity”) events. Manual coding evaluated stroke actions in three classes (i.e., forehand, backhand and serve), with additional descriptors of spin (e.g., slice). Movement data was classified according to the specific locomotion performed (e.g., lateral shuffling). The algorithm output for strokes were analysed against manual coding via absolute (n) and relative (%) error rates. Coded movements were grouped according to their frequency within the algorithm’s four movement classifications. Highest stroke accuracy was evident for serves (98%), followed by groundstrokes (94%). Backhand slice events showed 74% accuracy, while volleys remained mostly undetected (41–44%). Tennis-specific footwork patterns were predominantly grouped as “Dynamic” (63% of total events), alongside successful linear “Running” classifications (74% of running events). Concurrent stroke and movement data from wearable sensors allows detailed and long-term monitoring of tennis training for coaches and players. Improvements in movement classification sensitivity using tennis-specific language appear warranted.

## 1. Introduction

Historically, implementation of technology in tennis for detecting critical stroke and movement events have involved video coding methods or motion capture systems [1,2,3,4]. However, these processes have been laborious (i.e., manual notation of strokes) or cost-prohibitive (i.e., installation costs), limiting their integration in daily training environments. This has presented opportunities for wearable technology and machine learning approaches in sport, whereby data collected from sensors on an athlete are trained to detect the key “features” of the sensor output [5]. Accordingly, the continued development of these models in sport can benefit coaches and sports medicine staff to monitor athlete training loads and record sport-specific event data. In tennis, wearable sensors positioned on the hitting arm or racquet utilise accurate machine learning models for automated stroke detection [6,7] and present more affordable and accessible technological approaches to monitoring tennis training. However, their placement precludes quantification of running-based movement [7], which is also a critical component of tennis training and match-play profiles [8]. Single wearable sensors capable of interpreting both stroke and movement data would therefore be of undeniable value from a training monitoring perspective. Other sports have embraced the use of single cervical-mounted sensors (e.g., global positioning systems [GPS] and accelerometry) to report sport-specific event data [9,10,11,12,13,14], yet their efficacy in tennis remains largely untested.

Previous literature investigating the stroke detection accuracy from wrist- or racquet-mounted sensors have demonstrated classification accuracies >90% for serve, forehand and backhand stroke types [3,15,16,17]. Commercially available smart watches have been refined over time and classify these strokes with even greater accuracy (>95%) [15]. The classification of volleys though remains problematic even using wrist or multiple sensors [18], which is costly and impractical for players and practitioners [19]. To reconcile this issue, Perri et al. [20] validated a prototype algorithm from commercial cervically mounted GPS units and found 94%, 86% and 98% accuracy for detecting forehand “drive” (FH Drive), backhand “drive” (BH drive) and serves. Whilst these findings compared favourably with stroke detection accuracies of locally positioned sensors on the hitting arm [3,21], the overall technology represented a first iteration that excluded detailed movement analysis. The wearable sensor’s positioning at the cervical spine seems logical for reporting tennis’s bi-modal (i.e., hitting and moving) activity profiles and likely warrants further investigation.

Most commercial wearable sensors for sporting contexts are positioned at the cervical spine or trunk to infer whole-body mechanical demands [22]. This highlights an advantage compared to wrist-worn or racquet-mounted sensors, traditionally used in tennis, that report stroke events but provide limited insight into the locomotor demands of the sport. Whilst emerging evidence in tennis has utilised wrist-worn sensors and classify movement as “sprinting”, “running”, “walking” and “standing” activities [6], their validity is currently unavailable in the literature. Regardless, exploration of prototype machine learning algorithms from a single commercial cervically mounted wearable sensor to determine both stroke and movement events is currently missing. Thus, the aim of this study was to validate; (1) the stroke event detection algorithms and (2) a novel tennis movement detection algorithm from a wearable microsensor positioned at the cervical spine.

## 2. Materials and Methods

### 2.1. Participants

**Figure 1:**
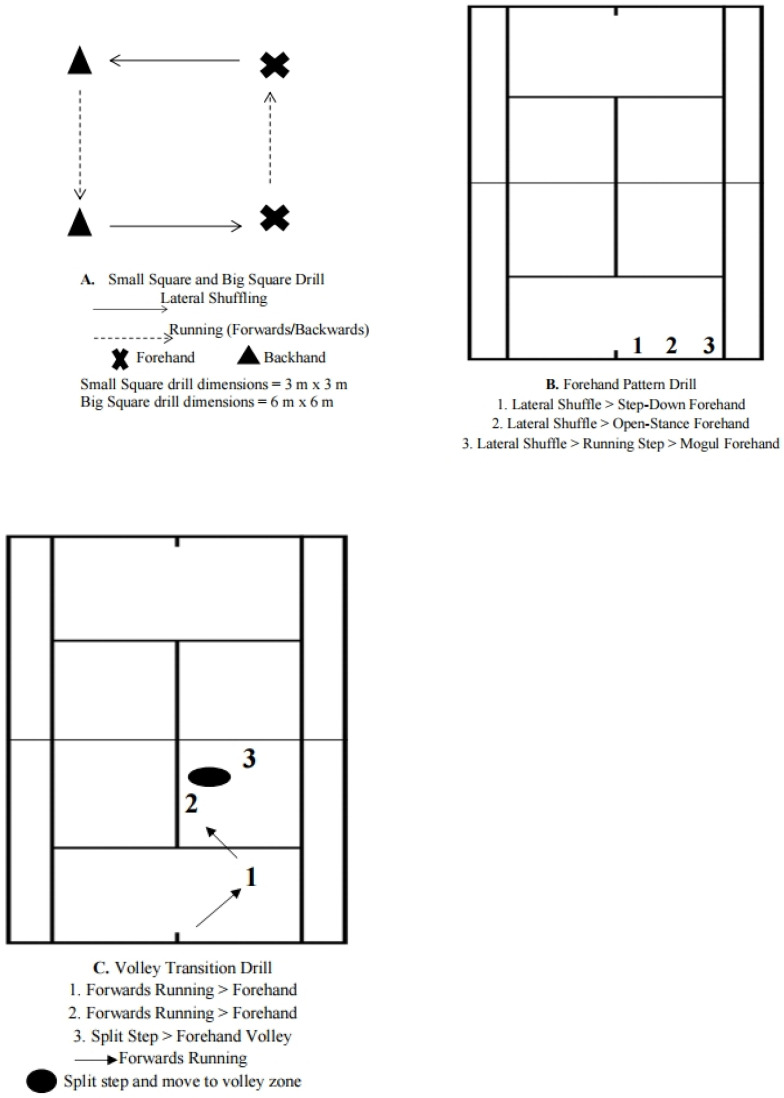
Illustrations of the four discrete movement drills used in the ‘simulated protocol’.

Data for the stroke validation component were originally collected in 2019 from 10 matches by eight junior-elite male tennis players (age 15.5 ± 1.6 y). The participating players were part of Tennis Australia’s high performance player pathway and engaged in ≈20 h of on-court tennis training per week. The players were also competing regularly in international level International Tennis Federation (ITF) tournaments. All players were right-handed with a double-handed backhand. Data for the movement validation were collected during respective ‘simulated’ and ‘natural’ tennis-specific movement drills (Figure 1). A healthy male tennis player (age 30 y) participated in the simulated tennis movement protocol comprising four discrete drills (Figure 1). A second healthy male (age 37 y) was involved in the data collection of the natural tennis movement protocol. Participants were previous competitors on the Association of Tennis Professionals (ATP) tour and experience as high-performance tennis coaches. The participants were right-handed and utilised a double-handed backhand. Both subjects provided their consent to participate in the study. Participants were familiarised with the movement drills by performing three trials of each drill at a self-determined ‘low’ and ‘high’ movement speed. All subjects and their legal guardians provided informed consent prior to participation in the study. The study methods conformed to the Declaration of Helsinki and was approved by the institutional Human Research Ethics Committee (ETH19-4062).

### 2.2. Stroke Validation

Matches used for stroke validation were conducted on hard and grass courts and played as a best-of-three sets match in accordance with governing body rulings [23]. Video cameras (HDR-CX700VE, Sony, Tokyo, Japan) were mounted on the fences surrounding the court and positioned 10 m above and 6 m behind the baseline as per previous filming of tennis training and match-play [24,25]. The wearable technology utilised to capture tennis stroke events as a commercial global positioning systems (GPS) unit (Catapult OptimEye S5, Catapult Sports, Melbourne, Australia), with an in-built triaxial accelerometer, gyroscope and magnetometer weighing 102 g. The device was worn in a neoprene harness provided by the manufacturer with the unit positioned in a pouch between the scapulae. Athletes were fitted for appropriate harness size to minimise movement on the skin [26] and thus, mitigate risk of noise in the raw data and artificial stroke detection.

Classification of stroke events were determined via a new prototype algorithm developed by the manufacturer (White Paper, Catapult Sports). Details of the algorithm are propriety of the manufacturer however, supervised random forest models are applied on the raw accelerometer, gyroscope and magnetometer data to classify strokes. These models have estimated overall accuracy to be 90% across “Serve”, “BH Drive”, “FH Drive” and “Other stroke” categories (White Paper, Catapult Sports). More specifically, these unpublished investigations have shown respective accuracies of 94%, 96.5% and 99.9% for FH drive, BH drive and serve events. Perri and colleagues [20] mostly confirmed these accuracies of 94%, 86% and 98% for detection of FH drive, BH drive and serve actions using a previous version of the algorithm. For the current study, raw accelerometer, gyroscope and magnetometer data from the 10 matches were processed using a new customised web-based application in the R Language (Rstudio, 1.1.463, Rstudio, Inc., Boston, MA, USA). The Coordinated Universal Time (UTC) (hh:mm:ss) was available for each stroke and re-analysed to compare against the previous dataset of Perri et al. [20].

This original dataset was manually notated by a coder with five years of experience coding tennis matches and a coefficient of variation (CV) of <2% for tennis training and match-play and 0.9% for stroke event classifications [20,24]. The dataset was first analysed to denote whether a stroke event was detected by the wearable device. This was then further scrutinised to classify whether the algorithm correctly identified the type of stroke (i.e., forehand, backhand or serve). In this example, a stroke labelled a “FH Drive” by the algorithm and manually coded as a forehand volley was considered to be correct from the algorithms perspective as it does not discriminate between stroke types beyond rally strokes. Instances where the algorithm detected a forehand, backhand or serve but classified it as an “Other stroke”, this was categorised as an incorrect classification. However, if “Other stroke” was recorded by the algorithm and a smash or stroke not meeting the previous criteria was played, this was considered to be correct.

The manually coded strokes were collated in the .csv file with algorithm stroke outcomes. Strokes were coded manually from the video footage in accordance with their basic type of stroke (i.e., forehand, backhand, serve) and further detailed by their specific spin or trajectory (i.e., rally, slice, volley, drop shot) (Table 1). Strokes that did not meet these general classifications (i.e., an underarm stroke to pass ball back to server) were coded as an “Other stroke” and treated separately. As the Catapult algorithm does not differentiate between smashes and serves, smashes were manually coded as an “Other stroke”. Racquet swings, which still resemble a forehand or backhand drive but without ball contact, were coded in respective “forehand” or “backhand” categories (Table 1).

### 2.3. Movement Validation

The simulated movement protocol is illustrated in Figure 1. The participant was familiarised with the drill requirements by the principal investigator and instructed to perform three trials of each drill at a self-determined ‘low’ and ‘high’ movement intensity (i.e., speed). Individual trials were separated by 30 s, where the participant remained stationary to minimise time alignment error in the raw data. Prior to commencement of each trial, the participant performed a backhand stroke that was used in the raw data trace to identify the start of each trial. The natural tennis movement protocol was developed based on terminology from previous research [28]. The participant was given instructions to move at ‘match-like’ intensities while performing all movements and stroke actions. A standardised rest period of 30 s was provided between trials to replicate the time-alignment procedures in the simulated movement protocol and closely simulate between point time during official tennis match-play.

The movement protocols in their entirety were recorded using video cameras (HDR-CX700VE, Sony, Japan) and positioned 10 m above and 6 m behind the baseline. Raw accelerometer, gyroscope and magnetometer data files were processed using a custom web-based application, which reported each detected movement action and the associated UTC. Four movement classifications existed from the prototype algorithm and are defined below as per the manufacturer (Catapult Sports, Melbourne, Australia):

- Alert Load = Preparatory movements preceding strokes (i.e., lowering centre of mass/racquet take back).
- Dynamic Load = ‘Explosive’ non-linear movements between strokes.
- Running Load = Linear running actions.
- Low Intensity Load = Walking actions.

Details of the algorithm to classify movement events are propriety of the manufacturer, though movement characteristics within the accelerometer are key in triggering specific classifications. The principal investigator reviewed the video footage and manually described the movement performed by the participant against the output from the prototype algorithm. Three members of the research team were provided with examples of given movements alongside their manual classification for verification.

### 2.4. Statistical Analyses

All cleaning of data and subsequent analysis was performed in both the R Language (RStudio, 1.1.463, RStudio, Inc.) and Microsoft Excel (Microsoft Excel, 16.49, Microsoft, Washington, DC, USA). There were 66 individual stroke events detected by the Catapult unit that were excluded from the analysis due to unresolved time-alignment error. For the stroke-level analysis, comparisons between the prototype algorithm and manual coding were performed via absolute and relative measures of error. Specifically, the number of events from the wearable sensor was divided by the total number of events in that category and multiplied by 100. Analysis was performed across basic stroke types (i.e., forehand, backhand, serve) and the detailed stroke classifications from Table 1. Movement event data are reported as a count across each category from the prototype algorithm and separated by protocol (i.e., simulated or natural).

## 3. Results

A total of 5094 stroke patterns were identified for analysis. Summary of forehand, backhand and serve stroke detection accuracies are reported in Table 2. Serves had the highest detection accuracies at 98%, with forehand and backhand reported at 94% accuracy. Similar non-detection and misclassification error rates (7–10%) were noted for both forehand and backhand swing events, with <1% non-detection and misclassification error on serve events. Overall false positive rates did not exceed 3% across the three stroke types. A total of 277 “Other stroke” events were detected, whereby 82 events were determined as false positives.

Table 3 reports the respective accuracies of forehand stroke types. Forehand “drive” events showed the highest overall accuracy of 95%, followed by forehand “block” stroke types (75%); however, the latter classification had a low overall occurrence (i.e., four total events). Forehand “dig” and “drop shot” strokes were unable to be detected by the prototype algorithm (accuracy = 0%). “Slice”, “volley” and “end range” forehand stroke types showed overall accuracies of 37–44%. Specifically for forehand volleys, the associated error was predominantly due to these strokes not being detected in the prototype algorithm (41% non-detection rate). Forehand “shadow” strokes had lower overall accuracy rates of 19%. “Smash” event detection accuracies are also reported in Table 3. There was a 2% difference in accuracy when smash events were considered correct as serve or other strokes (27% vs. 25%; Table 3).

Backhand-specific event detection accuracies are reported in Table 4. Backhand “drive” stroke types had the highest classification accuracy of 96%, followed by “slice” events (74% accuracy). Poorest detection accuracies were noted for backhand volleys, whereby 44% of these events were not detected by the prototype algorithm and resulted in an overall accuracy of 23%. The accuracy rates for detecting backhand “shadow” swings were greater than the forehand side, with an overall accuracy of 47%.

Data from the movement validation protocols are reported in Table 5. Within the simulated movement trials, “Alert Load” was mostly registered when the participant was lowering their centre of mass (n = 27). Alternatively, this movement classification from Catapult was least likely to be registered from split step actions (n = 7) or when the participant engaged in lateral shuffling (n = 2). For “Dynamic Load”, the highest proportion of movements detected in this category were adjustment steps (34%) followed by forwards running and lateral shuffling, which each contributed 15% of detected movements. Individual movement actions comprising the “Running Load” classification from Catapult were predominantly from forwards (n = 18) and backwards (n = 11) running actions. Lastly, “Low Intensity Load” events were mostly registered when the participant was performing lateral shuffling actions (56% of total “Low Intensity Load” events). Table 5 also contains data from the natural tennis movement protocol. Of the 41 total movement events registered from the prototype algorithm, 26 events were categorised as “Dynamic Load”. The second most detected movement category during the natural tennis movement trials was the “Low Intensity Load” classification, which registered a total of 12 standing actions.

## 4. Discussion

This study validated a tennis stroke detection and movement pattern recognition algorithm from a wearable sensor positioned at the cervical spine. The respective detection of accuracies of 98%, 95% and 96% for serve, FH Drive and BH drive strokes highlight the suitability of trunk-mounted wearable devices for quantifying hitting actions. However, the validity of the movement detection component of the algorithm was mixed for the different locomotor actions. These findings support the utilisation of trunk-mounted wearable sensor technology in tennis for monitoring of hitting demands [20], while signalling the opportunity for wearable sensors and their algorithm to better detect and classify sport-specific court-based movements.

Consistent with previous reports, the highest detection accuracy was observed for serve events [20]. Multiple racquet- and limb-mounted inertial sensors have previously achieved similar accuracies >95% [29]. This is presumably due to the serve being a closed skill and one with distinct roll features (detected by the gyroscope) similar to accuracies reported from cricket fast-bowling [30]. This has implications for both tennis coaches and medical staff given the importance of serving on the lumbar spine [31]. Accordingly, support staff members working in tennis can have confidence in implementing the present wearable sensor for longitudinal monitoring of serve volumes and their distribution to mitigate injury risk and optimise training exposure [32,33].

The stroke detection algorithm classified forehand, backhand and serve swing events with respective accuracies of 94%, 94% and 98%. In comparison to previous research [20], this shows a 5% improvement for classifying forehand swing events following recent manufacturer algorithm refinements. This may point to the trunk rotation signatures of the groundstroke actions being better reproduced. An alternative view may attribute these improves in accuracy towards the re-training the algorithm on a previously analysed dataset and thus, an overestimation of detection accuracy [19,34]. Despite this possible limitation, it remains likely that high accuracy classification rates for major strokes remain indicative of the unique trunk rotation and lateral flexion signatures registered from the gyroscope and accelerometer. This could also explain the low (≤3%) false positive rates from the present algorithm and further highlights its suitability for tennis stroke detection given the similarities with results from studies using wrist-worn devices [6].

Stroke detection performance declined for “slice” events, which concurs with reports in previous literature [6]. On the forehand side, this could be due to slice strokes being hit with highly variable ball speeds and therefore comparatively greater variation in upper limb and racquet kinematics [35], likely confounding the feature extraction and event classification. In a relative sense, backhand slice detection performed better than the forehand and may relate to more discernible trunk rotation in backhand slices and/or the higher frequency with which these shots are played. This would further support the notion of the magnitude and timing of trunk rotation as key features of interest, whilst explaining the difficulty of the wearable sensor’s position on the spine to accurately classifying volleys, given the negligible trunk movement in this stroke [36,37]. Similar degradations in volley detection accuracy also exist from wrist-worn sensors in samples of elite and sub-elite players, with precision rates of ≈70–80% [3,38]. The impact on tennis practitioners remain unclear though given volleys contribute <2% of strokes per match [2], yet are commonly featured in training drill prescription [25].

General classifications of locomotion revealed mixed results. Indeed, the algorithm’s specific “Running” classification captured instances of lateral shuffling and adjustment steps between the designed stroke events. This is interesting in the context of prior research that highlight the cyclical nature of running to result in more easily identifiable event detection from wearable sensors [39]. Therefore, it could be reasoned that the specific footwork actions of tennis are less easily separated from linear running activities when the sensor is placed on the cervical spine. It is unclear whether this stems from the methodology underpinning the algorithm’s development or an underlying limitation from the sensor’s placement at the trunk as distinct from more a distal orientation. In a similar vein, the algorithm classification of lateral shuffling as “Low Intensity” alongside walking would highlight opportunities for further model refinement with sport-specific contexts and terminology in mind.

Classifying tennis-specific footwork from previous wearable sensors worn at the shoes have achieved recognition rates of 63% that increased to 95% when higher proportions of training data are used in the model [40]. Whilst the present algorithm can be argued to resolve a practical issue regarding the use of multiple sensors, the ambiguous movement classifications from the manufacturer may limit practitioner use. For example, adjustment steps (i.e., preparing to start the stroke) common to tennis featured in both the “Alert” and “Dynamic” categories, which seems nebulous [7]. Indeed, that so much of a player’s court coverage was classified as “Dynamic Load” (63% of natural tennis protocol) may be traced back to subjectively “good” tennis movers, where the transition between individual steps of the movement cycle occur efficiently [41]. Alternatively, the notion that a trunk-mounted wearable sensor could adequately capture and identify the considerable number of tennis-specific footwork steps of previous research [28] seems ambitious if not unrealistic.

## 5. Limitations

A limitation of this study is that a small number of participants (n = 8) were involved. The homogenous sample of this study may represent a further limitation given a male-only cohort was included and future investigations on female participants are needed. Additionally, future research may wish to incorporate the influence of participant skill-level (i.e., amateur vs. professional) on algorithm performance. Another limitation of this study is that manipulation of the algorithm was not possible given it remains propriety of the manufacturer. Further, the stroke algorithm performance was assessed on the previous training dataset to allow further internal comparisons but may also contribute to the high accuracies in the present study. However, given the maintained accuracy from the previous investigation [20], it would appear the stroke detection algorithm remains suitable. Additionally, the outcome measures of accuracy in this study (“n” and “%”) may be considered a potential limitation. The authors also acknowledge that all manual coding of the stroke and movement data was performed by one analyst, where future studies could be strengthened by adding a second coder [42]. It is also acknowledged that our movement classifications involve a mixture of research and expert opinion, with no consensus, and thus influence our interpretation of the algorithm’s accuracy.

## 6. Conclusions

This study validated the stroke and movement detection algorithm using data from a commercial microsensor captured during tennis match-play and movement drills. Classification accuracies of 94%, 94% and 98% were observed for respective forehand, backhand and serve swing patterns and represents maintained detection rates from previous research. Small improvements were noted in this iteration of the algorithm, namely the improved backhand slice classification; however, volleys remained mostly undetected. The novel movement classifications show promise in their application, though may require adoption of sport-specific language to improve training of the algorithm for the user.

## Figures and Tables

**Table 1:** Manually coded stroke definitions.

Stroke Type	Definition
Drive	A typical ‘topspin’ or ‘flat’ forehand or backhand stroke. Also included ‘offensive’ lobs.
End-Range	A forehand or backhand stroke, typically played with the racquet arm at full stretch and in a wide position of the court.
Volley	A forehand or backhand stroke played ‘on-the-full’ with no bounce prior to the stroke.
Drop shot	A disguised forehand stroke that is played with the aim of the ball dropping short into the opposing player’s side of the court.
Block	A forehand or backhand stroke often played by the returner in response to a fast serve.
Slice	A forehand or backhand stroke played where the racquet’s forward-swing trajectory imparts backspin to the ball.
Dig	Strokes played with limited forward-swing and often are more vertical with a low to high ‘redirect’ trajectory.
Shadow	Any stroke pattern played in absence of a ball being contacted.

Adapted from Crespo and Miley [27].

**Table 2:** Stroke-Level Analysis of Catapult Detected Backhand Strokes vs. Manual Coding.

Stroke Type	Coded Events (n)	Catapult Events (n)	False Positives (n)	False Positive Rate (%) [False Positives/Catapult Events]	Non-Detected Strokes (n)	Non-Detection Rate (%) [Non-Detected Strokes/Coded Events]	Non-Classified Strokes (n)	Non-Classification Rate (%) [Non-Classified Strokes/Catapult Events]	Correctly Classified Strokes (n)	Correct Classification Rate (%) (Correctly Classified Strokes/Catapult Events]
Forehand	2142	1886	49	3%	179	8%	191	10%	1773	94%
Backhand	1936	1774	29	2%	136	7%	138	8%	1670	94%
Serve	1016	1032	3	0%	2	0%	2	0%	1012	98%

**Table 3:** Stroke-Level Analysis of Correctly Detected Catapult Forehand Strokes vs. Manual Coding Classifications.

Stroke Type	Catapult (n)	Coded Events (n)	Non-Detected Strokes (n)	Non-Detection Rate (%) [Non-Detected Strokes/Coded Events]	Non-Classified Strokes (n)	Accuracy (%) [Catapult/Coded Events]
Forehand drive	1640	1727	14	1%	73	95%
Forehand slice	31	72	23	32%	18	43%
Forehand volley	8	18	44	41%	16	44%
Forehand end range	80	218	69	32%	69	37%
Forehand drop shot	0	2	2	100%	0	0%
Forehand block	3	4	1	25%	0	75%
Forehand dig	0	1	0	0%	1	0%
Forehand shadow	8	42	21	50%	2	19%
Forehand lob	4	11	5	45%	2	36%
Smash (Serve as correct)	14	51	5	10%	32	27%
Smash (Other as correct)	13	51	5	10%	33	25%

**Table 4:** Stroke-Level Analysis of Catapult Detected Backhand Strokes vs. Manual Coding Classifications.

Stroke Type	Catapult (n)	Coded Events (n)	Non-Detected Strokes (n)	Non-Detection Rate (%) [Non-Detected Strokes/Coded Events]	Non-Classified Strokes (n)	Accuracy (%) [Catapult/Coded Events]
Backhand drive	1363	1423	19	1%	41	96%
Backhand slice	160	217	25	12%	32	74%
Backhand volley	14	62	27	44%	21	23%
Backhand end range	88	145	27	19%	30	61%
Backhand block	3	6	2	33%	1	50%
Backhand dig	3	6	2	33%	1	50%
Backhand shadow	36	77	30	39%	11	47%
Backhand lob	3	8	4	50%	1	38%

**Table 5:** Occurrence of manually coded movements within the Catapult classifications during “simulated” and “natural” tennis movement protocols. *“Simulated” movement protocol = (1) Small Square, (2) Big Square, (3) Forehand Pattern and (4) Volley Transition “Natural” movement protocol = (1) Gravity Split Step and Step-Down Forehand, (2) Split Step and Cross Step to Mogul Forehand, (3) Split Step and Cross Step to Arabesque Forehand, (4) Split Step to Drop Step and Defensive Forehand and (5) Off Forehand Transition Pattern*.

**Simulated Movement Protocol** **(n = 282)**			
**Alert Load** **(n = 89 [32%])**	**Dynamic Load** **(n = 90 [32%])**	**Running Load** **(n = 39 [14%])**	**Low Intensity Load** **(n = 64 [23%])**
Lowering Base (n = 27 [30%])	Adjustment Steps (n = 31 [34%])	Forwards Running (n = 18 [46%])	Lateral Shuffling (n = 36 [56%])
Adjustment Steps (n = 21 [24%])	Forwards Running (n = 15 [17%])	Backwards Running (n = 11 [28%])	Standing (n = 21 [33%])
Finishing Stroke (n = 16 [18%])	Lateral Shuffling (n = 15 [17%])	Lateral Shuffling (n = 8 [21%])	Forwards Running (n = 7 [11%])
Finishing Stroke and Lowering Base (n = 16 [18%])	Backwards Running (n = 9 [10%])	Adjustment Steps (n = 2 [5%])	
Split Step (n = 7 [8%])	Cross Step (n = 6 [7%])		
Lateral Shuffling (n = 2 [2%])	Split Step (n = 5 [6%])		
	Tennis Running Step (n = 5 [6%])		
	Finishing Stroke (n = 4 [4%])		
**Natural Tennis Protocol** **(n = 41)**			
**Alert Load** **(n = 2 [5%])**	**Dynamic Load** **(n = 26 [63%])**	**Running Load** **(n = 1 [2%])**	**Low Intensity Load** **(n = 12 [29%])**
Split Step and Adjustment Steps (n = 1 [50%])	Adjustment Steps (n = 6 [23%])	Cross Step and Lateral Shuffling (n = 1 [100%])	Standing (n = 12 [100%])
Split Step and Cross Step to Tennis Running Step (n = 1 [50%])	Split Step and Adjustment Steps (n = 6 [23%])		
	Cross Step (n = 3 [12%])		
	Split Step and Cross Step (n = 3 [12%])		
	Cross Step and Lateral Shuffling (n = 2 [8%])		
	Split Step and Cross Step to Tennis Running Step (n = 2 [8%])		
	Finishing Stroke (n = 1 [4%])		
	Split Step (n = 1 [4%])		
	Cross Step and Adjustment Steps (n = 1 [4%])		
	Lateral Shuffling and Adjustment Steps (n = 1 [4%])		

Data reported as absolute count of events (n) and the proportion of total events in each category (%).

## Data Availability

The data presented in this study are available on request from the corresponding author. The data are not publicly available due to privacy.

## References

[1] Pereira T.J.C., Nakamura F.Y., de Jesus M.T., Vieira C.L.R., Misuta M.S., de Barros R.M.L., Moura F.A. (2017). Analysis of the distances covered and technical actions performed by professional tennis players during official matches. J. Sports Sci..

[2] Whiteside D., Reid M. (2017). External match workloads during the first week of Australian Open tennis competition. Int. J. Sports Physiol. Perform..

[3] Whiteside D., Cant O., Connolly M., Reid M. (2017). Monitoring hitting load in tennis using inertial sensors and machine learning. Int. J. Sports Physiol. Perform..

[4] Gellard M., Jelcic M., Vial A. (2018). Using technology to improve practice and performance: A practical summary. ITF Coach. Sport Sci. Rev..

[5] Kautz T., Groh B.H., Hannink J., Jensen U., Strubberg H., Eskofier B.M. (2017). Activity recognition in beach volleyball using a deep convolutional neural network. Data Min. Knowl. Discov..

[6] Ganser A., Hollaus B., Stabinger S. (2021). Classification of tennis shots with a neural network approach. Sensors.

[7] Kramberger I., Filipcic A., Germic A., Kos M. (2022). Real-life application of a wearable device towards injury prevention in tennis: A single-case study. Sensors.

[8] Reid M., Cormack S.J., Duffield R., Kovalchik S., Crespo M., Pluim B., Gescheit D.T. (2019). Improving the reporting of tennis injuries: The use of workload data as the denominator?. Br. J. Sports Med..

[9] Reardon C., Tobin D.P., Tierney P., Delahunt E. (2017). Collision count in rugby union: A comparison of micro-technology and video analysis methods. J. Sports Sci..

[10] Hulin B.T., Gabbett T.J., Johnston R.D., Jenkins D.G. (2017). Wearable microtechnology can accurately identify collision events during professional rugby league match-play. J. Sci. Med. Sport.

[11] Roe G., Halkier M., Beggs C., Till K., Jones B. (2016). The use of accelerometers to quantify collisions and running demands of rugby union match-play. Int. J. Perform. Anal. Sport.

[12] Clarke A.C., Anson J.M., Pyne D.B. (2016). Proof of concept of automated collision detection in rugby sevens. J. Strength Cond. Res..

[13] Cummins C., Orr R. (2015). Analysis of physical collisions in elite national rugby league match play. Int. J. Sports Physiol. Perform..

[14] Kelly D., Coughlan G.F., Green B.S., Caulfield B. (2012). Automatic detection of collisions in elite level rugby union using a wearable sensing device. Sports Eng..

[15] Myers N.L., Kibler W.B., Axtell A.H., Uhl T.L. (2019). The Sony Smart Tennis Sensor accurately measures external workload in junior tennis players. Sport. Sci. Coach..

[16] Kos M., Zenko J., Vlaj D., Kramberger I. Tennis Stroke Detection and Classification Using Miniature Wearable IMU Device. Proceedings of the 23rd International Conference on Systems, Signals and Image Processing.

[17] Hadzic V., Germic A., Filipcic A. (2021). Validity and reliability of a novel monitoring sensor for the quantification of the hitting load in tennis. PLoS ONE.

[18] Pardo L.B., Perez D.B., Uranuela C.O. (2019). Detection of tennis activities with wearable sensors. Sensors.

[19] Rindal O.M.H., Seeberg T.M., Tjonnas J., Haugnes P., Sandbakk O. (2017). Automatic classification of sub-techniques in classical cross-country skiing using a machine learning algorithm on micro-sensor data. Sensors.

[20] Perri T., Reid M., Murphy A.P., Howle K., Duffield R. (2022). Validating an algorithm from a trunk-mounted wearable sensor for detecting stroke events in tennis. J. Sports Sci..

[21] Bulling A., Blanke U., Schiele B. (2014). A tutorial on human activity recognition using body-worn inertial sensors. ACM Comput. Surv..

[22] Crang Z.L., Duthie G.M., Cole M.H., Weakley J., Hewitt A., Johnston R.D. (2021). The validity and reliability of wearable microtechnology for intermittent team sports: A systematic review. Sports Med..

[23] ITF ITF Rules of Tennis. http://www.itf.tennis.com/about/organisation/rules.aspx.

[24] Perri T., Norton K.I., Bellenger C.R., Murphy A.P. (2018). Training loads in typical junior-elite tennis training and competition: Implications for transition periods in a high-performance pathway. Int. J. Perform. Anal. Sport.

[25] Murphy A.P., Duffield R., Kellett A., Reid M. (2014). A descriptive analysis of internal and external loads for elite-level tennis drills. Int. J. Sports Physiol. Perform..

[26] McLean B.D., Cummins C., Conlan G., Duthie G.M., Coutts A.J. (2018). The fit matters: Influence of accelerometer fitting and training drill demands on load measures in rugby league players. Int. J. Sports Physiol. Perform..

[27] Crespo M., Miley D. (1998). ITF Advanced Coaches Manual.

[28] Hughes M., Meyers R. (2005). Movement patterns in elite men’s singles tennis. Int. J. Perform. Anal. Sport.

[29] Yang D., Tang J., Huang Y., Xu C., Li J., Hu L., Shen G., Liang C.-J.M., Liu H. TennisMaster: An IMU-based online serve performance evaluation system. Proceedings of the 8th Augmented Human International Conference.

[30] McNamara D.J., Gabbett T.J., Blanch P., Kelly L. (2016). The relationship between variables in wearable microtechnology devices and cricket fast-bowling intensity. Int. J. Sports Physiol. Perform..

[31] Connolly M., Middleton K., Spence G., Cant O., Reid M. (2021). Effects of lumbar spine abnormality and serve types on lumbar kinematics in elite adolescent tennis players. Sport. Med. Open.

[32] Sombelon G.N., Myers N.L., Westgate P., Smith B.J., Kibler W.B. (2017). Tennis serve volume and its relationship to injury in professional women’s tennis players. J. Athl. Train..

[33] Myers N.L., Sciascia A.D., Kibler W.B., Uhl T.L. (2016). Volume-based interval training program for elite tennis players. Sports Health.

[34] Jowitt H.K., Durussel J., Brandon R., King M. (2020). Auto detecting deliveries in elite cricket fast bowlers using microsensors and machine learning. J. Sports Sci..

[35] Seeley M.K., Funk M.D., Denning W.M., Hager R.L., Hopkins J.T. (2011). Tennis forehand kinematics change as post-impact ball speed is altered. Sport. Biomech..

[36] Roetert E.P., Groppel J.L. (2001). Biomechanics of the volley. ITF Coach. Sport Sci. Rev..

[37] Crespo M. (1999). What tennis research tells us about…biomechanics of volleys and approach shots. ITF Coach. Sport Sci. Rev..

[38] Wu M., Fan M., Hu Y., Wang R., Wang Y., Li Y., Wu S., Xia G. (2022). A real-time tennis level evaluation and strokes classification system based on the Internet of Things. Internet Things.

[39] Willy R.W. (2018). Innovations and pitfalls in the use of wearable devices in the prevention and rehabilitation of running related injuries. Phys. Ther. Sport.

[40] Buthe L., Blanke U., Capkevics H., Troster G. A wearable sensing system for timing analysis in tennis. Proceedings of the 2016 IEEE 13th International Conference on Wearable and Implantable Body Sensor Networks (BSN).

[41] Giles B., Peeling P., Dawson B., Reid M. (2019). How do professional tennis players move? the perceptions of coaches and strength and conditioning experts. J. Sports Sci..

[42] Gastin P.B., McLean O.C., Breed R.V., Spittle M. (2014). Tackle and impact detection in elite Australian football using wearable microsensor technology. J. Sports Sci..

